# Monitoring Within-Person Dynamics in Adolescents Hospitalized for Acute Suicidal Distress: Protocol for an Intensive Longitudinal Study

**DOI:** 10.2196/93016

**Published:** 2026-09-25

**Authors:** Laura Nigro, Konstantin Drexl, Alberto Forte, Carole Kapp, Kerstin Jessica Plessen, Romain Gallien, Laurent Michaud, Marco Armando, Maude Schneider

**Affiliations:** 1 Clinical Psychology Unit for Intellectual and Developmental Disabilities Faculty of Psychology and Educational Sciences University of Geneva Geneva Switzerland; 2 Division of Child and Adolescent Psychiatry Department of Psychiatry Lausanne University Hospital and University of Lausanne Lausanne Switzerland; 3 Faculty of Biology and Medicine University of Lausanne Lausanne Switzerland; 4 Psychiatric Liaison Service Department of Psychiatry Lausanne University Hospital and University of Lausanne Lausanne Switzerland

**Keywords:** 3ST, actigraphy, adolescent psychiatry, ecological momentary assessment, IMV, inpatients, Integrated Motivational-Volitional Model, Interpersonal Psychological Theory of Suicide, IPTS, self-injurious behavior, suicidal ideation, suicide, Three-Step Theory

## Abstract

**Background:**

Adolescence represents a developmental period marked by heightened vulnerability to suicidal thoughts and behaviors (STBs), which remain a leading cause of death among youth, particularly psychiatric inpatients and after discharge. Contemporary ideation-to-action models conceptualize suicidal crises as dynamic processes driven by proximal psychological factors rather than static risk markers. While recent work has begun to examine these processes as they unfold in daily life, their integration into the clinical course of suicidal distress remains limited. From this perspective, relating short-term microlevel processes of STBs to their broader temporal changes across the transition of adolescents from acute inpatient hospitalization to early ambulatory aftercare constitutes an important area for further investigation.

**Objective:**

This protocol describes a multimodal, intensive longitudinal study examining the temporal unfolding of suicidal distress across complementary timescales. The integrative framework aims to identify prototypical trajectories of STBs from inpatient admission through the early postdischarge period. It further seeks to characterize heterogeneity in within-person processes central to ideation-to-action theories and STB-related affect regulation. Finally, the study aims to interrelate these microlevel dynamics of suicidal distress with its longitudinal trajectories at the macrolevel. Within this framework, the study examines how modifiable bioregulatory factors, including sleep and physical activity, as well as clinical process factors such as therapeutic alliance, are dynamically implicated across timescales.

**Methods:**

The study plans to recruit 100 adolescents hospitalized at a single site in French-speaking Switzerland. Within 72 hours of admission, participants start a first assessment period of up to 14 days, comprising 4 daily Ecological Momentary Assessment (EMA) surveys and continuous actigraphy recordings. A second assessment period follows participants for the 10 days after discharge and includes 2 daily EMA surveys. In addition, 4 in-person assessment sessions contextualize the intensive longitudinal time series with baseline clinical characteristics, diagnostic status, therapeutic processes, and episodic stressors. Planned analyses will use multilevel and latent class modeling approaches to identify longitudinal patterns of STBs, characterize between-person differences in within-person dynamics, and examine how these patterns relate to contextual factors.

**Results:**

Recruitment began on October 31, 2025, and is expected to continue until June 2028. The protocol was developed through the active integration of former patients’ perspectives and clinicians’ lived experience. Feedback from the first 5 participants supported the current implementation of the protocol.

**Conclusions:**

By focusing on the link between intraindividual short-term processes and clinical trajectories of STBs, this study will advance our understanding of how STBs and related psychological and behavioral processes interact over time during a critical period of distress. These insights aim to inform targeted, person-centered approaches to acute psychiatric care, offering better support for vulnerable adolescents transitioning from inpatient to outpatient care.

**International Registered Report Identifier (IRRID):**

DERR1-10.2196/93016

## Introduction

### Adolescent Suicidal Thoughts and Behaviors: A Major Public Health Concern

Adolescence is a key developmental period for the onset of suicidal thoughts and behaviors (STBs), conceptualized as a continuum including suicidal ideation, nonsuicidal self-injury (NSSI), suicide-related behaviors, and suicide attempts [1]. Accordingly, this paper uses STBs as an umbrella construct and the more specific term suicidal thoughts (STs) for thought-related findings. In Switzerland, a high-income country with a well-developed health care system, 14.7% of young people aged 15-24 years reported STs in 2022, and suicide remains the leading cause of death in this age group, with 14 deaths per 100,000 inhabitants [2]. Comparable trends have been observed internationally, with a large meta-analysis reporting high rates of STBs among youth across countries [3] and an Australian generational cohort showing increased STBs in more recent birth cohorts [4]. Adolescents requiring psychiatric hospitalization are particularly vulnerable, with suicidality affecting more than half of patients in several acute inpatient samples [5-7]. Furthermore, the highest suicide rates occur in the first month after discharge, with 484 deaths per 100,000 in this period [8-10]. Internationally, the COVID-19 pandemic further intensified psychological distress and demand for mental health services [11]. For example, a French study of over 20 million individuals aged 25 years or younger documented a marked postpandemic rise in psychiatric consultations and suicide-related hospitalizations, particularly among girls and adolescents aged 13 years and older [12].

Such findings highlight the growing vulnerability of adolescents, the burden of STBs, and the resulting pressure on mental health services to provide adequate care and support [13].

### Rethinking Traditional Approaches to Inpatient Suicidal Distress

Traditionally, research aiming to understand STBs has been primarily grounded in distal and nonmodifiable indicators such as psychiatric diagnoses, prior STBs, and demographic characteristics [14-16]. Yet, available meta-analytic evidence indicates that these risk-factor-based approaches have limited predictive value, with little to no improvement over the past 50 years [17-19], while standardized risk-assessment tools display minimal prognostic accuracy in clinical settings [20,21]. Although structured screening and assessment of STBs remain important components of clinical care, their role should be distinguished from prediction-oriented risk stratification [22]. Concerns have been raised about using risk scales or global risk categories as the primary basis for treatment or discharge decisions, as such practices may foster a false sense of security or contribute to reductive or coercive care [23,24]. Accordingly, current National Institute for Health and Care Excellence (NICE) [25] guidance emphasizes comprehensive psychosocial assessment, individualized formulation, and attention to the person’s needs and safety over static risk categorization [26].

Reflecting this shift, recent perspectives increasingly move beyond conceptualizing suicidality in terms of stable risk factors, instead emphasizing its time-sensitive and dynamic nature as it unfolds across multiple timescales and clinical transitions [27-29]. Consistent with this view, contemporary evidence indicates that short-term intraindividual variability in suicidal ideation (within hours or across days), rather than its peak severity, may be a more accurate indicator of acute short-term suicidal behavior [30-32]. Research should therefore move beyond single-time-point detection of STBs toward characterizing their emergence, persistence, and reduction over time.

This is particularly important during hospitalization for STBs, to better adapt care to adolescents’ dynamic and individualized trajectories of distress associated with STBs [33].

In line with this shift toward dynamic conceptualizations, 3 contemporary theoretical models—the Interpersonal Theory of Suicide [34,35], the Integrated Motivational-Volitional Model (IMV) [36,37], and the Three-Step Theory (3ST) [38]—offer key insights into the development of STs and the transition to suicidal behaviors. Each model describes the ideation-to-action process through different proximal intrapersonal and interpersonal factors, including painful representations of the self in relation to one’s life and identity (eg, defeat, entrapment, and hopelessness) or to others (eg, thwarted belongingness, perceived burdensomeness, and connectedness) [39,40]. All 3 theories emphasize that ST stems from thwarted belongingness, perceived burdensomeness, and hopelessness. The IMV model further incorporates defeat and entrapment, while the 3ST focuses on the interplay between hopelessness and psychological pain, particularly when the latter overwhelms connectedness. The 3 models converge on the assumption that the volitional step toward suicidal behavior is shaped by an individual’s capability for suicide, characterized by low fear of death, high pain tolerance, and access to means. The IMV framework also identifies impulsivity as facilitating this transition [34,37,38,41].

Importantly, these dynamic processes may remain active during hospitalization and after discharge, even after the most acute phase of a suicidal crisis has subsided. Theoretical accounts also suggest that ST may function as a short-term escape from aversive internal states when other regulatory strategies are insufficient, potentially contributing to their persistence or recurrence [42,43]. Studying hospitalization and the early postdischarge period is therefore essential for understanding persistence of and recovery from suicidal distress. However, empirical research has struggled to capture these temporal dynamics, largely due to methodological limitations.

### Toward New Methodologies: Ecological Momentary Assessment in Adolescent Suicide Research

#### Overview

A key limitation of the existing empirical literature is that most has relied on cross-sectional studies or designs with long assessment intervals (weeks, months, or years) [44]. Since these approaches are poorly suited to the highly dynamic nature of STBs [18], Ecological Momentary Assessment (EMA) has emerged as a promising method for studying suicidal processes in adolescents’ daily lives [30,45]. Using frequent smartphone-based assessments of internal states, behaviors, and situational factors, EMA captures moment-to-moment psychological mechanisms and STB-related fluctuations as they unfold in daily life. Beyond research, EMA may also be integrated into clinical practice by informing monitoring tools during hospitalization, supporting ambulatory follow-up, and serving as an adjunct to targeted interventions [46,47].

In this context, concerns have been raised about the potential iatrogenic effects of routinely asking about STBs; however, no empirical data to date indicate any harm from such momentary assessments [48]. Moreover, current evidence supports the feasibility and acceptability of daily ecological assessments in adolescents, including clinical samples hospitalized for severe STBs [49-51].

#### Longitudinal Profiles of STB Dynamics

One major benefit of this ecological momentary paradigm is its ability to investigate STB-related processes across multiple timescales. At the macro level, this allows examination of whether STBs follow distinct longitudinal patterns across different phases of the care pathway (Figure 1). In one seminal intensive longitudinal study [52], adolescents’ EMA reports revealed distinct profiles based on the daily frequency of STs and the severity of ST-related urges: *persistently low, declining,* and *persistently high* profiles. Comparable profiles emerged independently in inpatient and postdischarge samples, although the same adolescents were not followed across the transition between these care contexts. Conceptually similar low, declining, and persistently elevated trajectories have emerged across prospective studies with diverse populations, assessment schedules, and follow-up periods, including adolescent inpatient and posthospitalization samples [45,53], adults receiving treatment for depression [54], and individuals with a first episode of psychosis [55]. Complementary recent work has examined temporal features of STBs using intensive multimodal or longer-term prospective designs, with distinct aims, including prediction of subsequent STBs [56] and identification of subtypes or trajectories of suicidal ideation associated with later suicide attempts [57].

Despite emerging evidence, important gaps remain in understanding how longitudinal STB trajectories unfold within the same adolescents across transitions in care and how they relate to the broader clinical and contextual environment. Their temporal characteristics also remain insufficiently understood, particularly the timing, direction, and rate of change in STs and broader STBs during acute clinical transitions.

**Figure 1:**
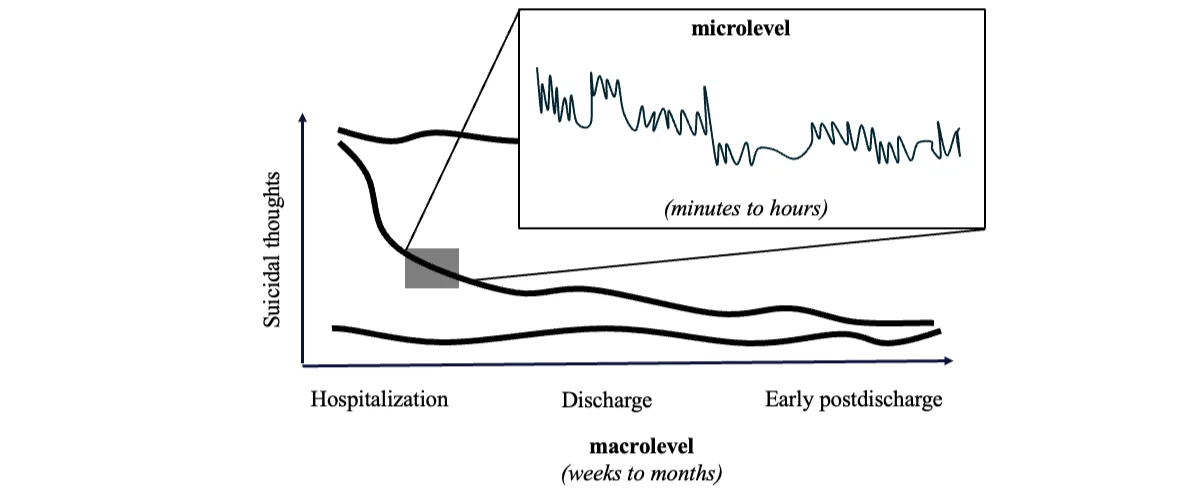
Multiscale dynamics of suicidal distress during the transition from hospitalization to early aftercare.

#### Moment-to-Moment Processes and STBs

Zooming in from broader trajectories to fine-grained temporal patterns, EMA also captures moment-to-moment processes that may shape suicidal distress (Figure 1). By capturing psychological processes as they unfold in daily life, EMA allows researchers to examine microlevel mechanisms emphasized in ideation-to-action models, namely, how STBs are triggered, fluctuate, and are regulated in response to internal and interpersonal proximal factors. Importantly, these insights are clinically relevant, as effective coping with STBs often hinges on recognizing and addressing such rapidly shifting proximal processes.

Posthospitalization intensive longitudinal studies have revealed meaningful within-day associations between STs (frequency, duration, and intensity) and factors such as perceived burdensomeness, thwarted belongingness, and connectedness, whereas evidence for next-day associations has been less consistent [58-60]. Interaction effects have nevertheless emerged when low connectedness co-occurs with high burdensomeness or high hopelessness, predicting more severe same-day and, to some extent, next-day STs [58]. These findings demonstrate the value of intensive longitudinal measures, although evidence remains scarce and inconsistent, particularly among hospitalized adolescents [44].

Such inconsistencies likely stem from methodological variability (eg, differing assessment windows), selective emphasis on particular model components, and limited alignment between theoretical constructs and their operationalization in EMA protocols, resulting in an incomplete empirical picture [60]. Between-person differences in within-person processes can manifest, for instance, in the degree to which aversive emotional states trigger STBs and the extent to which aversive states diminish following momentary STBs. Recent idiographic findings in adolescents similarly suggest that dynamic associations may vary substantially across individuals despite weak or absent group-level effects [61]. Such heterogeneity has recently been observed in hospitalized adult men [62], challenging both the generalizability of previous group-level findings in adolescents [63] and the universality of hypotheses drawn from ideation-to-action theories and affect-regulation frameworks [62].

Throughout this protocol, *microlevel dynamics* refer to concurrent, lagged within-day, and day-to-day associations within the same adolescent, whereas *macrolevel trajectories* refer to broader changes in STBs across hospitalization and early postdischarge follow-up (Figure 1). A pivotal next step is therefore to link individual differences in microlevel dynamics with macrolevel longitudinal profiles to better understand how the theorized processes contribute to the clinical trajectory of adolescents experiencing suicidal distress. The following sections extend our framework to include bioregulatory factors of sleep, physical activity, and circadian rhythms, as well as therapeutic factors that may act as putative moderators across timescales.

STs can vary on complementary temporal scales. At the macrolevel, trajectories of suicidal distress may follow persistently high, rapidly declining, or persistently low patterns across hospitalization and early aftercare. At the microlevel, suicidal distress may fluctuate over minutes to hours through changes in STs, affective states, interpersonal appraisals, and regulatory processes. The figure illustrates that these short-term within-person dynamics may be characteristic of broader clinical trajectories.

### The Role of Sleep, Physical Activity, and Circadian Rhythms

Adolescents hospitalized for suicidal crises experience complex interactions between sleep, physical activity, and circadian rhythms that may modulate STBs (Figure 2). Emerging evidence from intensive longitudinal research suggests that these 3 domains, particularly during the sensitive transition from hospitalization to ambulatory care, may play distinct but interrelated roles in influencing proximal psychological processes such as feelings of social defeat or anhedonia.

Sleep disturbances, including insomnia and nightmares, have consistently been shown to co-occur with and precede STBs in adolescents [64-66]. Acute sleep loss and delayed bedtimes frequently precede suicide attempts [67]. Recent longitudinal studies suggest that poorer sleep quality and shorter-than-usual sleep duration predict higher next-day STs through increased negative affect, emotional reactivity to interpersonal events [68], and anhedonia [69]. These findings suggest that sleep problems may influence the ideation-to-action transition through aversive psychological states (eg, defeat, entrapment, and hopelessness) [70], dysfunctional cognitive styles (eg, rumination) [71], and behavioral factors (eg, impulsivity) [72].

Physical activity also relates to mental health outcomes in hospitalized adolescents. Observational studies among adolescent psychiatric inpatients found that physically active individuals experience a lower likelihood of suicide attempts than inactive peers [73,74]. Qualitative research indicates that adolescents perceive exercise as a valuable strategy for mood regulation and coping during hospitalization [75] when barriers are adequately addressed [76]. Randomized controlled trials demonstrated that structured exercise programs improve mental health outcomes [77], with one study also reporting benefits for STB recovery in adults [78]. Emerging research on potential mechanisms highlights homeostatic regulation of stress responses [79] and corrective psychological experiences that enhance self-esteem [80]. Nevertheless, the within-person relationship between physical activity and momentary STBs, and its specific role within the ideation-to-action framework, remains insufficiently understood.

Circadian rhythms orchestrate sleep and physical activity and shape the temporal dynamics of STs [81]. Across the lifespan, longitudinal studies identify several circadian risk markers, including an evening chronotype, misalignment between internal rhythms and external schedules, and irregular sleep-wake patterns [82-84]. Furthermore, suicidal behaviors show diurnal patterns, with most attempts occurring in the early morning hours [85]. By tracking within‑person variability in the sleep-wake cycle, researchers can examine these fluctuations in relation to microlevel STB-related mechanisms and macrolevel changes across this clinical transition. Collectively, these factors represent an important yet understudied aspect of the suicidal process during hospitalization and early aftercare. Intensive longitudinal research using EMA and actigraphy holds considerable promise for elucidating dynamic interactions among these factors and may inform targeted interventions in adolescent suicide prevention.

**Figure 2:**
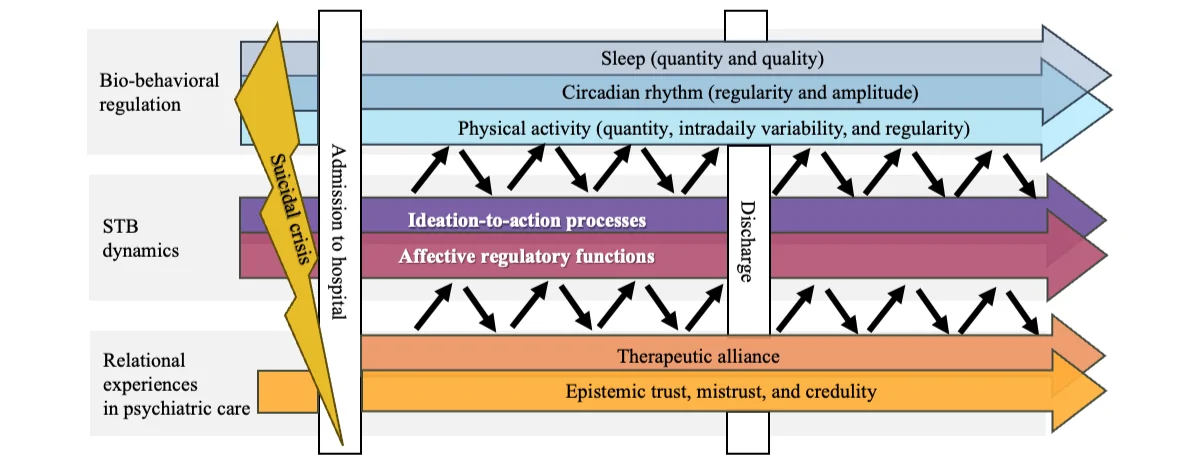
Dynamic integration of modifiable targets during acute hospitalization and early aftercare. The figure depicts 3 domains of interest that may dynamically interact during the transition from inpatient to ambulatory care: biobehavioral regulation, suicidal thought and behavior (STB)–related psychological dynamics, and relational experiences in psychiatric care. These domains represent interrelated processes situated within psychiatric care.

### The Subjective Therapeutic Relationship and Epistemic Trust

Therapeutic processes during hospitalization are pivotal for crisis stabilization and longer-term outcomes but may affect STBs through heterogeneous mechanisms and timescales (Figure 2) [86]. In adolescent suicidal distress, the subjective therapeutic relationship is particularly relevant because it may intersect with interpersonal representations central to ideation-to-action models, such as thwarted belongingness, perceived burdensomeness, and social disconnection [35,37,38]. However, the timescale over which these processes impact STBs remains unclear, as prior research has predominantly focused on between-person outcomes [86-88]. During this period of intensive clinical intervention, treatment not only addresses acute symptoms but may also shape the recovery trajectory as adolescents transition to ambulatory care, when STBs can be particularly pronounced [58,89]. The potential benefits of interactions with the interdisciplinary clinical team depend on patients’ willingness and ability to perceive interpersonally transmitted information as both reliable and relevant [90]. This capacity, known as epistemic trust, is a cornerstone of the corrective interpersonal experience fostered by the therapeutic relationship [91]. A robust therapeutic alliance may enhance reciprocal trust [88], facilitating positive shifts in STBs by allowing patients to rebuild trust in their human environment and counteract negative representations of social relations central to the theorized ideation-to-action process. Conversely, epistemic stances of mistrust or excessive credulity may underlie maladaptive cognitive and affective processes and sustain painful interpersonal representations within ideation-to-action processes, such as perceived burdensomeness [92]. Despite their potential relevance, little is known about how therapeutic alliance and epistemic trust relate to STB dynamics during hospitalization and the transition to ambulatory care.

### Building an Integrative Framework From Hospitalization to Ambulatory Care

The transition from hospitalization to early ambulatory care can be conceptualized across complementary temporal scales. Broader trajectories of suicidal distress unfold across days and clinical phases, whereas more proximal fluctuations in affective, interpersonal, and STB-related processes may occur over minutes to hours (Figure 1). Within this multiscale perspective, the present framework focuses on selected modifiable domains relevant to hospitalization and early aftercare: biobehavioral regulation, STB-related psychological dynamics, and relational experiences in psychiatric care (Figure 2). The interplay between aversive states and STBs may follow individually distinct patterns, reflecting person-specific regulatory functions of STBs in alleviating unbearable psychological pain. Sleep, physical activity, and circadian rhythms represent modifiable factors that may buffer momentary negative psychological states, influencing short-term STB dynamics. Concurrently, adolescents’ subjective experience of the therapeutic relationship, particularly epistemic trust within the therapeutic alliance, may shape corrective social learning processes. Beyond prediction alone, the study of dynamic processes increasingly supports a shift toward an individualized, process-based understanding of suicidal distress, emphasizing person-specific patterns of regulation and meaning [29]. Integrating these complementary perspectives through intensive longitudinal methodologies such as EMA and actigraphy may capture between-person differences in within-person dynamics and connect previously separate research targets.

### The Present Study

#### Overview

Building upon this integrative framework, this study examines the dynamic experiences of adolescents in suicidal crises throughout hospitalization and during their immediate transition to outpatient care. This protocol describes a multimodal and intensive longitudinal study designed in close collaboration with clinicians and young partners. The study is guided by 5 interrelated aims ranging from the characterization of broad clinical trajectories to fine-grained within-person processes and their contextual moderators. Although STBs are conceptually linked within the suicidal process and both constitute relevant study outcomes, suicidal behaviors are expected to occur much less frequently during the monitoring period. Consequently, the intensive longitudinal analyses are primarily designed to characterize within-person fluctuations in STs and related motivational processes. Suicidal behaviors will nevertheless be systematically recorded to allow exploratory examination on a descriptive and between-person level.

#### Aim 1: Characterize Macrolevel Trajectories of STBs From Hospital Admission to Early Ambulatory Care

This first aim conceptually extends previous work that identified *persistently high*, *persistently low*, and *declining* profiles of STs [52]. Unlike prior studies, this project will follow the same participants throughout hospitalization and the initial days of subsequent ambulatory care, allowing us to explore the continuity and change of longitudinal profiles within the same adolescents. It will also investigate baseline variables (eg, STB severity, comorbidities, and trait measures of ideation-to-action constructs). Beyond testing whether previously identified trajectory profiles will conceptually replicate, this study examines whether longitudinal trajectories persist or change across discharge from inpatient care, providing a unique opportunity to investigate this important clinical transition from a within-person perspective.

#### Aim 2: Examine Within-Person Microlevel Dynamics of STBs and Ideation-to-Action Constructs

The second aim focuses primarily on the momentary dynamics of STs by examining their reciprocal temporal associations with the ideation-to-action constructs assessed through EMA (ie, thwarted belongingness, perceived burdensomeness, connectedness, hopelessness, defeat, entrapment, and psychological pain). When sufficient instances of suicidal behavior are observed, exploratory models will examine whether preceding STs are associated with subsequent suicidal behavior and whether this transition varies as a function of impulsivity and acquired capability for suicide. These analyses may provide preliminary insight into ideation-to-action transitions but are not intended as a definitive test of these transitions.

#### Aim 3: Link Microlevel Dynamics to Macrolevel STB Trajectories

The third aim is to examine the micro-macro correspondence, defined here as the association between individual differences in the short-term within-person associations described in Aim 2 and broader longitudinal STB trajectories from hospitalization to early postdischarge follow-up described in Aim 1. Specifically, this study will examine whether the strength and direction of momentary short-term associations predict longitudinal profile membership. In addition, it will examine dynamic indicators of STB variability as potential predictors of profile membership.

#### Aim 4: Examine Sleep, Physical Activity, and Circadian Rhythms Across Micro- and Macrolevel STB Processes

The fourth aim extends the framework to modifiable bioregulatory factors. Sleep, physical activity, and circadian rhythms will be examined both as predictors or features of macrolevel STB trajectories and as momentary or daily predictors and moderators of within-person STB-related processes. We expect that more regular sleep-wake patterns and higher physical activity will be associated with persistently low or declining STB trajectories and with weaker coupling between aversive states of negative affect and ideation-to-action constructs and STBs.

#### Aim 5: Examine Therapeutic Alliance and Epistemic Stance in Relation to STB Dynamics

The fifth aim addresses therapeutic relational processes across hospitalization and the transition to ambulatory care. Specifically, therapeutic alliance and epistemic trust, mistrust, and credulity will be examined in relation to both STB trajectories and within-person processes. We expect stronger therapeutic alliance and greater epistemic trust to be associated with weaker links between negative interpersonal representations and STBs and a higher likelihood of persistently low or declining STB trajectories. The corresponding results may clarify whether and how adolescents’ perceived therapeutic relationship within a multidisciplinary team of care providers is associated with changes in STBs.

Together, these 5 aims examine how psychological, bioregulatory, and therapeutic processes may interact across complementary timescales to influence STBs in vulnerable adolescents.

## Methods

### Ethics Statement

This study adheres to the Declaration of Helsinki [93] and was approved by the Cantonal Commission for Ethics in Human Research of the Canton of Vaud (Commission cantonale d’éthique de la recherche sur l’être humain, CER-VD; study ID: 2025-00752) on July 23, 2025.

### Participants and Recruitment

This single-site study will recruit 100 adolescents hospitalized for an acute mental health crisis in French-speaking Switzerland. The recruiting psychiatric inpatient unit for adolescents at Lausanne University Hospital (Unité d’hospitalisation psychiatrique pour adolescents) accommodates over 200 admissions each year. Patients are considered for recruitment based on the following criteria: (1) presence of STB as evaluated by the psychiatrists performing the initial psychiatric status assessment at admission, (2) age between 14 and 18 years at enrollment, and (3) agreement for their outpatient therapist and parents or legal guardian to be informed about their participation. Exclusion criteria are (1) insufficient knowledge of French to understand the study materials and (2) presence of an intellectual disability. Patients who enter the recruiting unit multiple times during the data collection phase may participate multiple times in the study. These cases will allow us to assess the stability of STB-related dynamics across multiple episodes in a single-case design. However, the specific procedures and analyses planned for these cases are beyond the scope of this paper, which focuses on initial participation.

Participants will be recruited consecutively as part of routine clinical care. During daily clinical meetings, clinicians and research collaborators assess the eligibility of patients admitted within the past 48 hours. In parallel with these proactive invitations, patients receive study flyers in their welcome booklet upon admission and may themselves express interest in participating, prompting an evaluation of the eligibility criteria at the next clinical meeting. When eligibility is confirmed, a study collaborator approaches the respective patient later that day to explain the research project and deliver the informed consent procedure. Once signed informed consent has been received, a kickoff session is scheduled within 24 hours. According to Swiss legislation [94], adolescents aged 14 years and older are legally permitted to provide written informed consent without parental assent, provided they are deemed capable of judgment. Given the importance of close collaboration between the clinical team and parents during hospitalization and because data collection transitions to the home environment, parents systematically receive a dedicated information letter, and the research team remains available for questions about the data collection protocol.

### Study Procedure

After obtaining informed consent, a study collaborator extracts relevant sociodemographic and routine medical data and schedules 2 baseline assessment sessions (Figure 3). During the kickoff session, participants are provided with an actigraphy wristband and undergo individual training in EMA data collection, during which the full set of EMA items is presented. Additionally, they complete a STB interview and self-report measures. A subsequent psychiatric diagnostic interview assessing Axis-I disorders of the *Diagnostic and Statistical Manual of Mental Disorders, 5th edition* is scheduled flexibly. During hospitalization, participants complete 4 daily EMA surveys for up to 14 days on a study smartphone configured for this purpose, while continuously wearing the actigraphy device. This monitoring period may be shorter depending on length of stay. Before discharge, the STB interview is repeated, along with the measures of therapeutic alliance and epistemic trust. During the following 10-day outpatient monitoring phase, participants respond to 2 daily EMA surveys and continue actigraphy recordings. This reduced sampling was chosen to minimize participant burden and avoid interference with school and other daily routines. A final round-up session is held in person or via secure video call, during which participants complete the STB interview and a retrospective interview about contextual stressors that might have influenced the monitoring period. Participants return the actigraphy device and receive a CHF 100 (US $124) voucher. In addition, they may opt to receive invitations to regularly held participatory board meetings. During recruitment, participants are invited to discuss possible obstacles to study-related smartphone use, including parental or residential caregiver restrictions. At the round-up session, participants are systematically asked whether any practical barriers interfered with EMA participation, including phone restrictions, confiscation, loss of access, technical issues, or competing obligations.

**Figure 3:**
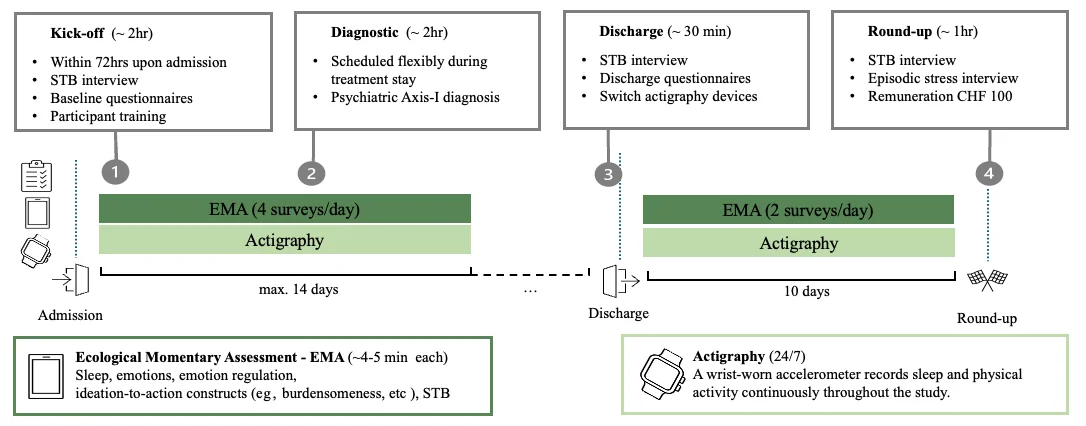
Longitudinal assessment protocol. Actigraphy devices are changed to mitigate risk of data loss. EMA: Ecological Momentary Assessment; STB: Suicidal Thoughts and Behaviors.

### Measures

#### Sociodemographic and Clinical Routine Data

Information on sociodemographic variables such as age, gender, family, and educational status, as well as clinical context variables, including referral (eg, acute STs, suicide attempt, and number of hospitalizations), is drawn from the clinical administrative patient record system. Study collaborators also extract scores from the French version of the Health of the Nation Outcome Scale for Children and Adolescents rated by clinicians at intake [95,96]. Patient record data are manually transferred to the electronic case report form in REDCap (Vanderbilt University). Sociodemographic variables that remain missing (eg, gender identity) are complemented during the kickoff session.

#### Comprehensive STB Interview

Consistent with the continuum-based conceptualization of STBs [1] and with previous studies [72], we assess STBs using the Columbia-Suicide Severity Rating Scale (C-SSRS) [97]. Because the C-SSRS provides limited coverage of NSSI, complementary items selected from the Self-Injurious Thoughts and Behaviors Interview-Revised [98,99] and the Clinical Interview for Borderline Personality Disorder for Adolescents [100] were added to ensure a comprehensive STB evaluation at the kickoff, discharge, and round-up sessions.

#### Trait-Level Self-Report Measures

Baseline self-report measures include symptom severity screening scales for prodromal psychotic symptoms, depression, generalized anxiety, alcohol and substance use, and sleep disturbances (Table 1). Psychometric scales also cover theoretical ideation-to-action constructs, therapeutic process constructs, and traits related to rest-activity profiles, including chronotype and physical activity habits. All self-report measures were selected based on previous use in research, evidence of psychometric quality, availability in French, and brevity. All scales provide one or more composite scores, with higher scores representing higher symptom severity or stronger expression of traits, respectively. In addition, symptom severity scales offer validated cutoffs to identify clinically significant symptomatology for the respective disorder. For measures unavailable in French, instruments were translated by a professional translator. A backward translation by the research team was conducted to identify and discuss inconsistencies and reach consensus on a final translation.

**Table 1:** Trait-level self-report measures.

Assessment target		Items, n	Instrument name
**Symptom severity/screening scales**			
	Depressive symptoms	9	Patient Health Questionnaire (PHQ-9) [101,102]
	Anxiety symptoms	7	Generalized Anxiety Disorder Scale (GAD-7) [103,104]
	Borderline personality disorder	11	Borderline Personality Functioning Scale for Children and Adolescents (BPFS-C11) [105,106]
	Prodromal psychotic symptoms	16	Prodromal Questionnaire-16 (PQ-16) [107,108]
	Alcohol and substance use	3-7	French Screening for Problematic Alcohol and Drug Use in Adolescents (DEP-ADO) [109]
**Ideation-to-action constructs**			
	Impulsivity	20	Short Urgency-Premeditation-Perseverance-Sensation Seeking-Positive Urgency Impulsivity Scale (UPPS-P) [110,111]
	Hopelessness	20	Beck Hopelessness Scale (BHS) [112,113]
	Suicidal thoughts and behaviors related coping	12	Efficacy To Cope With Suicidal Thoughts And Urges Scale (ECSTUS) [114]
	Feelings of defeat and entrapment	8	Social Defeat and Entrapment Scale (SDES) [115]
	Thwarted belongingness and burdensomeness	15	Interpersonal Needs Questionnaire (INQ) [41,116]
	Psychache	3	Unbearable Psychache Scale (UP3) [117]
	Social connectedness	8	Social Connectedness Scale Revised (SCS-R) [118]
	Acquired capability for committing suicide	20	Acquired Capability for Committing Suicide Scale (ACCS) [116,119]
**Therapeutic process constructs**			
	Epistemic trust	15	Epistemic Trust Mistrust and Credulity Questionnaire (ETMCQ) [120,121]
	Therapeutic alliance	8	Health Alliance Questionnaire (HAQ-8) [122]
**Modifiable lifestyle factors**			
	Sleep disturbances	8	PROMIS Pediatric Short Form v1.0-Sleep Disturbance 8a (PROMIS-Ped-SleepDis-8a) [123]
	Chronotype	8	Munich Chronotype Questionnaire (MCTQ) [124,125]
	Physical activity	4-7	International Physical Activity Questionnaire-Short Form (IPAQ-SF) [126]

#### Diagnostic Interviews

The diagnostic baseline assessment includes a comprehensive interview for Axis-I psychiatric disorders using the Kiddie Schedule for Affective Disorders and Schizophrenia [127,128].

#### Retrospective Stress Interview

During the round-up session, the Youth Life Stress Interview [129,130] is conducted to contextualize the intensive longitudinal data by exploring the timing and intensity of stressful life events experienced by each participant during the study protocol. This semistructured interview systematically examines domains hypothesized to be transient stressors for adolescents (eg, academic, peer, and family-related stress). For each reported event, the interviewer collects the adolescent’s narrative. Two independent raters subsequently categorize each narrative according to predefined characteristics (eg, life domain, whether the event was interpersonal, and the degree to which the event depended on the adolescent’s actions) and quantify its severity on a 9-point Likert scale. Any discrepancies between raters are resolved through discussion, with consultation from senior researchers when necessary. The rate of initial disagreement will be quantified to assess interrater reliability. The interview manual underwent prior in-house translation into French for this study.

#### EMA Protocol

In line with current restrictions on personal phone use during hospitalization, patients receive a study-specific smartphone configured exclusively for the study’s EMA surveys, with all other apps and websites blocked. During home visits and the postclinic period, participants use their personal smartphones. EMA surveys are accessible through a web link sent by SMS for each EMA assessment. The delivery of trigger SMS messages and EMA surveys is managed through the principal REDCap project database and survey tools [131,132] hosted on a secure institutional server. Delivery of EMA surveys follows a semirandom schedule, with notifications sent at randomly selected times within fixed time windows (eg, morning period: 8 AM-10 AM; noon period: 12 PM-2 PM; afternoon period: 4 PM-6 PM; evening period: 7 PM-9 PM), accommodating the ward’s weekly agenda. Two additional reminders are sent after 10 and 20 minutes if the participant does not respond, and the questionnaires remain accessible for a total of 1 hour. If a participant fails to respond to 4 consecutive EMA surveys, a research team member reaches out via the communication channel initially indicated by the participant. For the postclinic monitoring phase, the morning EMA is delivered within individually scheduled time windows, whereas the second EMA prompt is primarily delivered between 4:00 PM and 5:30 PM, unless the participant is systematically unavailable, in which case the delivery window is postponed.

The EMA surveys, both during and after hospitalization, include 35 items assessing aspects such as momentary STs, capability for suicide, NSSI, affective and regulatory processes, and intra- and interpersonal processes, plus a 7-item sleep diary (Table 2). Based on the initial participants, the median response duration is approximately 5 minutes for morning surveys and 4 minutes for the remaining surveys. Items were selected from prior EMA studies investigating adolescent STBs, complemented by items from the Experience Sampling Methodology Item Repository, and refined through discussions with clinicians, adolescents, and the research team to ensure clinical relevance and feasibility (see the full item list in Multimedia Appendix 1).

**Table 2:** Ecological Momentary Assessment survey sections.

Section	Items, n
Sleep (once daily)	7^a^
Interpersonal context and motivation	4
Affect states	5
Emotion regulation	3
Interpersonal factors	6
Intrapersonal factors	8
NSSI^b^	2
STB^c^	6
STB-related coping	1

^a^Only administered at the first active response of each day.

^b^NSSI: nonsuicidal self-injury.

^c^STB: suicidal thought and behavior.

#### Actigraphy

During both monitoring phases, participants wear a GENEActiv wristband (ActiveInsights Inc; CE certified) equipped with a triaxial accelerometer, a lux-sensor (dynamic range of 3-48 lux), and a thermistor (0-60 °C). Wrist-level acceleration is recorded at 10 Hz to preserve battery life. Raw sensor data will be extracted and preprocessed with the *GGIR* package [133,134] using its heuristic sleep detection algorithm [135]. Major sleep periods detected by this algorithm will be revised based on the sleep diary items from the EMA surveys, as well as on stereotypic patterns in the light and temperature signals [136]. Target indicators include measures of sleep quantity (eg, total sleep time), timing (eg, Sleep Midpoint and Sleep Regularity Index), and quality (eg, sleep efficiency). We will quantify physical activity in terms of volume, intensity, and timing [137,138]. Furthermore, circadian regularity and rhythmicity will be assessed using nonparametric measures of fragmentation [139,140] as well as cosinor-based rhythmometry [141]. Actigraphy adherence and nonwear will be evaluated systematically.

At discharge, participants will switch actigraphy wristbands to mitigate risk of data loss. Nonwear periods will be identified using an external detection procedure based on characteristic changes in acceleration combined with rate-of-change in device temperature [136]. In addition, participants are asked during the predischarge and round-up sessions whether they removed the device for extended periods and, if so, why. These reports will be used to identify and address sources of nonwear.

### Involvement of People With Lived Experience

This study protocol was designed through a participatory process involving multiple forms of exchange between researchers, patients, and clinicians. Clinicians from the inpatient unit and researchers held multiple interdisciplinary meetings, and researchers relocated their workplace to the unit to foster attention to the clinical setting and mutual exchange during the preparatory phase. Additionally, former patients were involved as young partners during and after their treatment stay, reviewing the initial research questions, study protocol, and materials. For instance, young partners were specifically consulted on the EMA item catalogue, including item selection, perceived repetitiveness, and expected completion burden. Their feedback supported retaining the overall EMA survey length as acceptable in light of the study aims. Based on their input, we implemented an open, voluntary format for participatory advisory board meetings, allowing young partners to join or leave as they wish and ensuring clear communication about the study’s progress and findings.

### Crisis Response Management

Our ethical considerations focused on ensuring that participants find adequate assistance if they experience acute STBs during data collection. Participants will be informed that their responses to EMA surveys are not monitored live. Based on expert consensus [142] and consultations with young partners from the recruitment site, the crisis-response procedures adopt a proportionate, nontransgressive stance that encourages adolescents to seek support while preserving their agency.

Predefined EMA responses indicating intense STs or suicidal behavior automatically activate a message within REDCap. During hospitalization, the message encourages participants to contact the inpatient clinical team immediately or request contact from clinical staff. After discharge, it directs participants to a trusted person, their outpatient health care provider, the relevant psychiatric on-call and emergency services, and the project telephone line. Research collaborators monitor survey delivery and completion during office hours, but do not continuously review individual responses. Four consecutive missed surveys prompt contact for technical or adherence support and are not independently interpreted as evidence of clinical deterioration. The project telephone line is available on weekdays during office hours for participants who would like additional support from someone familiar with the study. Calls that cannot be answered immediately are returned at the next available opportunity, whereas messages received outside office hours are returned on the following working day. Participants are informed that this line does not replace emergency or routine clinical services. After discharge, responsibility for ongoing clinical care remains with the participant’s outpatient clinicians and the relevant emergency services. Before discharge, the research team records the contact details of the clinician responsible for ambulatory follow-up and sends them an information letter; parents or legal guardians are also informed of study participation at enrollment.

STBs are additionally assessed during structured interviews and may also be disclosed during participant-initiated calls, video sessions, or other direct contacts. In these situations, trained study collaborators use the Columbia Risk Identification protocol alongside clinical judgment to determine whether further psychosocial assessment or immediate intervention is required, in line with general clinical practice and NICE guidelines [25], and the clinical pathways of the inpatient unit [143]. Current STs accompanied by intent and either a detailed plan or preparatory behavior trigger immediate escalation. During hospitalization, the psychiatrist on duty is contacted. Outside the hospital, the collaborator remains in contact with the participant while contacting the psychiatric on-call service. For participants younger than 18 years, parents are also informed when acute risk requires intervention. Confidentiality is maintained except when disclosure to parents or health care professionals is necessary to secure urgent assistance.

These procedures are communicated transparently during enrollment. They are intended to balance participant autonomy and well-being while ensuring that proportionate clinical assistance is available when significant suicidal distress arises.

### Statistical Analysis

#### Analytic Overview

Statistical analyses will be organized according to the 5 study aims. Macrolevel analyses refer to longitudinal patterns of STBs across hospitalization and the early postdischarge period. Microlevel analyses refer to short-term within-person associations across repeated EMA assessments and will therefore be restricted to the hospitalization phase, during which 4 assessments are collected per day. Primary analyses will include only participants’ initial study participation.

#### Aim 1: Macrolevel Trajectories of STBs

Extended mixed models using latent classes and latent processes [144,145] will be used to estimate clusters of individuals with similar STB trajectories. This modeling approach accommodates individually irregular and unbalanced time series, including variation in hospitalization duration. With respect to the difference in EMA sampling between the 2 study phases, we will estimate longitudinal profiles for the hospitalization phase and the early aftercare period separately. Continuity and change of profiles between the 2 study phases will be described using a confusion matrix.

Model selection will consider Akaike Information Criterion, Bayesian Information Criterion, convergence, entropy, posterior classification probabilities, class size, and clinical interpretability. A solution will be considered adequate for subsequent class-based analyses when entropy is ≥0.80, the mean posterior probability of assignment is ≥0.70 in each class, and the smallest class includes at least 5% of the sample and no fewer than 5 participants. Smaller or less clearly separated classes may be reported descriptively when clinically meaningful. If no solution meets these criteria, we will explore alternative modeling approaches, such as latent growth models [146], considering their respective assumptions and constraints. The absence of an adequate class structure will be interpreted as evidence regarding the contextual and methodological boundary conditions of previously reported trajectory profiles.

Multinomial logistic regression models will subsequently examine whether profile membership is associated with baseline variables (eg, severity of STBs, comorbidities, and trait measures of ideation-to-action constructs). Sensitivity analyses will examine associations of profile membership with hospitalization length and EMA compliance.

#### Aim 2: Microlevel Dynamics

Multilevel mixed-effects models will examine concurrent, lagged within-day, and day-to-day associations between STs and the momentary ideation-to-action constructs specified in Aim 2. At this stage, all the momentary ideation-to-action constructs specified above will be considered, while their precise operationalization (eg, as single items or aggregated composite scores), combination in the analyses, and analytic treatment will depend on sufficient within-person variability, data completeness, and analytical feasibility. Lagged analyses will examine whether fluctuations in these constructs are associated with subsequent STs and whether STs are associated with subsequent changes in these constructs. Models will include random intercepts and, where supported by the data, random slopes to capture between-person variation in within-person associations. Suicidal behaviors will be explored if sufficient events are observed.

These analyses will be restricted to the hospitalization phase. Postdischarge EMA data will primarily contribute to longitudinal trajectory analyses and complementary daily-level analyses rather than analyses requiring the same density of within-day observations. Statistical power simulations indicate that variation in random slopes can be estimated robustly with sample sizes as small as 60 participants, with acceptable precision at even lower sample sizes [147] (see Multimedia Appendix 1 for the power analysis).

#### Aim 3: Correspondence Between Micro- and Macrolevel Processes

To examine whether individual differences in microlevel dynamics correspond to broader longitudinal trajectories, person-specific random-effects estimates from the multilevel models will be entered as predictors of profile membership in multinomial logistic regression models. These analyses will be interpreted in light of classification uncertainty in the latent-profile solution. For dynamic indicators of person-level STB variability as predictors of longitudinal profile membership, we will compute an a priori set of intraindividual dynamics including (1) instability as indicated by the root-mean-square of successive differences, (2) rigidity as indicated by the lag1-autocorrelation, (3) overall fragmentation based on the fragmentation index [148], and (4) average time-to-momentary-recovery [149]. We will consider conceptual and empirical overlap among these indicators when interpreting their associations with longitudinal profile membership [150,151].

Inferential class-based analyses will be conducted only when the adequacy criteria specified for Aim 1 are met. If an adequate class structure is not identified, class-based micro-macro analyses will be considered descriptive or will be replaced by exploratory analyses relating microlevel parameters to continuous macrolevel summaries, such as phase-specific mean STB intensity, presence of suicide attempts and longitudinal change.

#### Aim 4: Sleep, Physical Activity, and Circadian Rhythms

Sleep, physical activity, and circadian indicators will be examined at the temporal level appropriate to each construct. At the macrolevel, indicators such as sleep efficiency, sleep timing and regularity, acceleration profiles [138], and the activity balance index [139] will be examined as predictors of longitudinal STB profiles. At the microlevel, time-varying indicators will be examined as predictors of STBs and as moderators of the within-person associations investigated under Aim 2.

Additionally, we will examine physical activity during the 30-minute period preceding an EMA assessment as a lagged predictor of STBs and ideation-to-action constructs. Fluctuations in sleep will be examined as predictors of momentary and daily changes in daytime affective functioning and ideation-to-action constructs using a unified dynamic structural equation modeling framework [152].

#### Aim 5: Therapeutic Alliance and Epistemic Stances

Therapeutic alliance and epistemic trust, mistrust, and credulity will be examined across the relevant temporal levels. At the macrolevel, baseline levels and changes across hospitalization will be tested as predictors of longitudinal STB profiles. At the microlevel, these constructs will be examined as moderators of associations between aversive psychological states, interpersonal experiences, and subsequent STBs. These analyses will be considered secondary because the therapeutic measures are collected less frequently than the EMA variables.

#### Exploratory Analyses of Suicidal Behaviors

As suicidal behaviors are expected to be infrequent during the monitoring period, analyses of these outcomes will be explicitly exploratory. Depending on the observed number of events, analyses may compare participants who report at least one suicidal behavior with those reporting none with respect to their clinical characteristics and microlevel motivational dynamics. No definitive conclusions will be drawn regarding the transition from ideation to action when event counts are insufficient for appropriately adjusted models.

#### Data Completeness, Missingness, and Data Quality

Overall and phase-specific EMA compliance, completion times, consecutive missed prompts, excluded careless responses, and the number of usable lagged predictor-outcome pairs will be reported. To control for potential biases in nonresponse, missing data will be examined in relation to study phase, time since enrollment, time of day, clinical severity, STB intensity, and documented contextual barriers (eg, reported phone restrictions).

Predefined indicators of potentially careless responding, including extremely short completion times and extreme-response-mode patterns, will be applied following previously proposed thresholds [139]. Depending on indicator strength, affected observations will either be excluded a priori or examined through sensitivity analyses.

Mixed-effects models will use all available observations with complete data for the predictor-outcome pair and covariates under examination. Although these models accommodate unequal numbers of observations across participants, they do not remove bias arising from informative missingness; consequently, the missingness analyses described above will inform interpretation of the findings [141].

For analyses requiring daily physical-activity distributions, actigraphy nonwear periods will be imputed using person-specific averages from comparable periods defined by time of day, weekday vs weekend, and hospitalization vs aftercare. Sensitivity analyses will repeat the principal models after excluding days with substantial nonwear.

#### Statistical Inference and Multiple Testing

Hypothesis tests involving overlapping predictors and outcomes will be grouped a priori into families corresponding to the principal analytic blocks: (1) baseline and person-specific indicators of ST dynamics as predictors of longitudinal-profile membership; (2) momentary psychological predictors and moderators of STBs; (3) sleep, physical activity, and circadian indicators; and (4) therapeutic alliance and epistemic-stances analyses. Within each family, the family-wise error rate will be controlled at α=.05 using Holm’s sequentially rejective procedure.

Both unadjusted and Holm-adjusted *P* values will be reported alongside effect estimates and 95% CIs. For Bayesian analyses, highest density intervals will also be interpreted at 90% when using informed priors and at 95% when using weakly-informed default priors. Analyses outside these prespecified families, including rare suicidal behaviors and repeated study participation, will be explicitly identified as exploratory.

## Results

Study preparation included discussions with the clinical team and patients who volunteered as young partners. Upon ethical approval, a pilot phase with 5 participants was conducted to allow final adaptations to the clinical reality of this field research. The planned procedures were implemented as intended: scheduled assessment sessions were completed, participants were able to access and respond to EMA prompts on the study smartphones, actigraphy monitoring was successfully conducted, and no major practical barriers related to smartphone use or study procedures were identified. Informal verbal feedback from participants was also generally favorable. Accordingly, no major protocol modifications were deemed necessary, and the 5 pilot participants could therefore be retained in the final study sample.

As of July 2026, 40 eligible patients had been invited since October 31, 2025, of whom 30 had agreed to participate, corresponding to an acceptance rate of 75%. Of these, 29 participants have been enrolled; 1 consenting patient could not participate because both available study smartphones were in use. The resulting recruitment rate of approximately 3.4 participants per month suggests that the target sample will be reached around May or June 2028. This estimate is conservative because recruitment is currently limited to 3 days per week and 2 study smartphones. Recruitment coverage and device availability can be increased if required to maintain the planned timeline.

Although the pilot observations support feasibility, our expectations for EMA response compliance are mixed. Prior EMA studies in adolescent suicidality indicate that missingness can be substantial, although direct comparison is limited by differences in design, duration, population, and setting [153,154]. In contrast, a recent EMA study in hospitalized adolescents [155] supports a more optimistic expectation of high response compliance and low participant burden. Recent meta-analytic evidence indicates that compliance rates vary substantially, even after accounting for typical design parameters such as study length and number of items [156]. We therefore anticipate that the implemented protocol is feasible, while acknowledging uncertainty in the expected compliance range, estimated at approximately 50%-80%. Based on previous actigraphy studies in adolescent samples, we expect a moderate proportion of nonwear-related missingness, approximately 10%-17% of the monitoring period on average [157,158], with potential differences between the structured inpatient setting and the less controlled aftercare phase.

## Discussion

### Overview

By linking fine-grained psychological and physiological data with adolescents’ lived experiences of acute suicidal crises, this protocol proposes a dynamic, multiscale framework that integrates contemporary theoretical perspectives with intensive longitudinal methods and is designed to address important methodological and clinical gaps.

### Expected Implications: Enhancing the Identification and Clinical Approach to Suicidal Distress

#### Deepening Insight Into Suicidal Distress

The present project aims to advance the field’s understanding of suicidality not as a static “risk profile,” but as a dynamic process unfolding across multiple timescales and clinical transitions [27,28,159]. To our knowledge, this is the first study to combine EMA and actigraphy to investigate the temporal course and proximal-dynamic predecessors of STBs in psychiatrically hospitalized adolescents from admission through the first 10 days after discharge. By jointly capturing moment-to-moment psychological, biobehavioral, and relational processes alongside broader STB trajectories across hospitalization and early aftercare, the study may identify patterns and mechanisms that contribute to or relieve suicidal distress in real-life contexts. This high-resolution, multiscale perspective may move beyond one-off risk estimations toward a more time-sensitive and personalized understanding of vulnerability [33].

#### Toward a Context-Sensitive and Personalized Understanding of Suicidal Distress

This protocol is embedded within a clinical landscape shifting toward process-based and person-centered approaches to suicidal distress. This evolution is reflected in the current NICE guidelines [25], which emphasize comprehensive psychosocial assessment and individualized understanding rather than reliance on standardized risk stratification alone. In French-speaking Switzerland, the “RENCONTRE” framework similarly emphasizes the temporal and contextual unfolding of suicidal distress and a collaborative clinical stance. Both frameworks emphasize acknowledging suicidal distress without assuming that crises require immediate resolution or coercive intervention, instead advocating for proportionate responses that balance clinical needs with collaboration, youth empowerment, and respect for autonomy [25,160]. We acknowledge that this orientation reflects a specific cultural and normative context subject to ongoing debates [22] and does not imply rejecting structured assessment, clinical decision-making, or protective interventions when required. Rather, it emphasizes integrating these practices with adolescents’ subjective experiences, needs, and autonomy. This protocol reflects this balance by combining standardized documentation of STB severity and change with an idiographic understanding of each participant’s clinical trajectory. This grounding in clinically oriented frameworks strengthens the ecological validity and translational relevance of the study by linking the processes examined to the complexity of adolescent suicidal distress encountered in clinical practice.

Capturing how psychological and physiological processes evolve from hospitalization through early postdischarge care may also inform future stepped-care strategies in which care intensity is adapted to patients’ changing needs. Although this study is observational and does not deliver an intervention, EMA and actigraphy provide structured means of capturing fluctuations that are poorly assessed retrospectively and may ultimately inform monitoring approaches or ecological momentary interventions [161]. Within person-centered frameworks such as RENCONTRE, these tools could complement therapeutic interactions by supporting a shared understanding of fluctuations over time and positioning adolescents as active participants in making sense of and navigating their own recovery [162]. At this stage, our contribution is to demonstrate the potential value of an idiographic, context-sensitive paradigm for generating evidence to support a more personalized and collaborative understanding of suicidal distress [163].

### Strengthening Research Relevance Through Patient-Partner Involvement

In line with recent paradigms in participatory research, the present project has been shaped by a commitment to patient and public involvement (PPI). This perspective acknowledges patients’ experiential knowledge as a distinct and complementary form of expertise, alongside clinical and scientific knowledge. It also resonates with national priorities for improving the quality and safety of health care in Switzerland, as emphasized in its national report on health care quality and health care safety [164], and with recommendations from the Swiss Federal Office of Public Health to strengthen the voice of patients and caregivers and foster mutual learning between professionals and service users. Within research, PPI offers several benefits: it helps ensure that study questions address issues of real concern, methods remain acceptable and feasible, and outcomes are meaningful and relevant to those most directly affected. Realizing these benefits requires research teams to develop the necessary competencies and draw on available guidance to support meaningful collaboration with patient partners [165].

Beyond these general principles, PPI played a specific and formative role in the development of this project. Adolescents from the inpatient unit participated in reviewing the initial research questions, the intensity of monitoring, and the acceptability of specific procedures. Their feedback directly informed key design decisions, including ensuring that intensive longitudinal assessments (ie, EMA and actigraphy) would remain feasible and nonintrusive during a period of acute suicidal distress; shaping the communication style of study materials to avoid triggering, paternalistic, or overly technical formulations; and refining crisis-response messages in EMA surveys so that they align with the unit’s clinical posture and the lived experience of youth in crisis. These contributions ensured that the protocol remained sensitive to the realities of adolescents hospitalized for acute suicidal distress and responsive to the constraints of a psychiatric ward environment.

### Limitations and Future Directions

Despite its innovations, our protocol has some limitations. First, the study prioritizes a selective set of modifiable and repeatedly measurable domains that can be assessed intensively during hospitalization and early postdischarge follow-up. It therefore does not provide a fully integrative account of suicidal recovery. Important influences such as family interactions [166], trauma history [167], broader social and environmental adversity [168], and additional physiological dimensions [169] are not directly integrated into the present protocol and should be considered in future extensions of this work.

A further limitation concerns the relatively sparse sampling of EMA observations, which increases the impact of missing data. This limits the detectable granularity of the within-person processes, since rapid fluctuations below the timescale of 1 hour are systematically missed. Similarly, adherence to both EMA and actigraphy protocols may vary across participants, further affecting data completeness. In addition, the postdischarge follow-up period, though capturing a critical period for recovery from suicidal crisis, remains relatively short and provides limited insight into macrolevel trajectories. This limited duration, however, was a deliberate trade-off: by restricting follow-up to 10 days post discharge, we prioritized feasibility and minimized participant burden, ensuring higher acceptability of the protocol in this population.

Despite frequently repeated assessments, analyses involving longitudinal profile membership (Aim 1) and downstream between-person analyses (Aims 3-5) remain based on approximately 100 independent participants. Allowing for 20%-30% dropout, this sample approaches those of previous implementations of the same latent process approach [52]. However, in the absence of transferable methods, we could not ground our sample size planning on relevant aspects of model performance, such as recovering the correct number of classes, estimating class-specific trajectories without bias, or classifying individuals accurately. Therefore, statistical power remains uncertain for the proposed latent profile estimation, and downstream between-person analyses may be underpowered.

Furthermore, person-specific random effects are estimated with varying precision depending on time-series length and completeness. Treating them as observed predictors does not propagate this uncertainty and may attenuate associations and produce overly precise estimates [151]; the corresponding associations based on estimates from microlevel and macrolevel models will therefore be considered exploratory.

Finally, findings from adolescents hospitalized for acute mental health crises may not generalize to the broader population of adolescents experiencing STBs. In addition, as with all mixture-model approaches, latent profile solutions may vary across samples and modeling specifications; therefore, trajectory classes will be interpreted as heuristic summaries of longitudinal heterogeneity rather than discrete natural categories.

Looking ahead, future work should examine the feasibility of extending intensive longitudinal assessments into routine care and longer-term recovery, including the potential use of idiographic models, tailored feedback, and real-time monitoring in clinical decision-making.

### Conclusion

By linking granular psychological and physiological data to the lived experience of adolescents in acute crisis, our project advances a dynamic, multiscale approach to better understanding individual suicidal distress. It contributes to emerging models of personalized and temporally responsive care, with the potential to transform how suicidality is understood, monitored, and supported across critical clinical transitions. Beyond improving prediction, this work lays the foundation for future steps toward more responsive, context-aware interventions that reflect the complexity of youth suicidal distress. As such, it represents an important step toward informing future efforts to reduce burden, stigma, disability, and long-term consequences associated with suicide.

## Appendix

Multimedia Appendix 1 Ecological momentary assessment items and power analysis for the planned multilevel models.

## Abbreviations

3ST: Three-Step Theory
C-SSRS: Columbia-Suicide Severity Rating Scale
EMA: Ecological Momentary Assessment
IMV: Integrated Motivational-Volitional Model
NICE: National Institute for Health and Care Excellence
NSSI: nonsuicidal self-injury
PPI: patient and public involvement
ST: suicidal thought
STB: suicidal thought and behavior

## Data Availability

No data are available at this stage, as this manuscript reports a study protocol. Once data collection is completed, individual-level research data will not be made publicly available because of the highly sensitive nature of the information collected, particularly data concerning suicidal thoughts and behaviors. To support scientific transparency and reproducibility while preserving participant privacy and confidentiality, sample-level summary statistics, including correlation matrices, may be made available where appropriate.
