# Supplementary material for: Monitoring Within-Person Dynamics in Adolescents Hospitalized for Acute Suicidal Distress: Protocol for an Intensive Longitudinal Study
Source: JMIR Res Protoc. 2026 Sep 25;15:e93016. doi: 10.2196/93016 (PMC13653208; doi:10.2196/93016)
Supplement: Supplementary file 1 [file resprot_v15i1e93016_app1.docx]

This is a Multimedia Appendix to the article “**Monitoring Within-Person Dynamics in Adolescents Hospitalized for Acute Suicidal Distress: Protocol for an Intensive Longitudinal Study”** published in the Journal of Medical Internet Research – Protocols (JMIR Protocols). For full copyright and citation information, see http://dx.doi.org/10.2196. 93016

Laura Nigro^1,2^, Konstantin Drexl^1,2^, Alberto Forte^1^, Carole Kapp^1^, Kerstin Jessica Plessen^1^, Romain Gallien^1^, Laurent Michaud^1^, Marco Armando^1^ & Maude Schneider^2^

^1^ Division of Child and Adolescent Psychiatry, Department of Psychiatry, Lausanne University Hospital and University of Lausanne, Lausanne, Switzerland.

^2^ Clinical Psychology Unit for Intellectual and Developmental Disabilities, Faculty of Psychology and Educational Sciences, University of Geneva, Geneva, Switzerland.

**Corresponding Authors**

Laura Nigro, 40, Boulevard du Pont d’Arve - 1205 Genève, +41223799579, [laura.nigro@unige.ch](mailto:laura.nigro@unige.ch)

Konstantin Drexl, 40, Boulevard du Pont d’Arve - 1205 Genève, +41223799579, [konstantin.drexl@unige.ch](mailto:konstantin.drexl@unige.ch)

# Ecological Momentary Assessment Items

| **Questions / items** | **Response format** |
| --- | --- |
| **Sleep and circadian rhythms** | |
| À quelle heure t'es-tu mis.e au lit ? | Heure : ☐☐ Minute : ☐☐ |
| À quelle heure as-tu essayé de t'endormir ? | Heure : ☐☐ Minute : ☐☐ |
| En combien de temps t'es-tu endormi.e ? | Heure : ☐☐ Minute: ☐☐ |
| À quelle heure t'es-tu réveillé.e avant de te lever ? | Heure : ☐☐ Minute : ☐☐ |
| À quelle heure t'es-tu levé.e pour commencer ta journée ? | Heure : ☐☐ Minute : ☐☐ |
| J'ai bien dormi cette nuit. | Pas du tout 0 ──── 100 Très fortement |
| Je me sens reposé.e ce matin. | Pas du tout 0 ──── 100 Très fortement |
| **Current context** | |
| Où suis-je ? | ☐ À l’UHPA ☐ À la maison ☐ À l’école ☐ Dans les transports publics ☐ À un autre endroit : __________ |
| En ce moment, je suis… | ☐ Seul.e ☐ Avec quelqu’un |
| Je me sens à l’aise en compagnie de cette/ces personne.s. | Pas du tout 0 ──── 100 Très fortement |
| Je préférerais être seul.e. | Pas du tout 0 ──── 100 Très fortement |
| **Current emotional state** | |
| En ce moment, je me sens… heureux.se | Pas du tout 0 ──── 100 Très fortement |
| En ce moment, je me sens… triste | Pas du tout 0 ──── 100 Très fortement |
| En ce moment, je me sens… honteux.se | Pas du tout 0 ──── 100 Très fortement |
| En ce moment, je me sens… détendu.e | Pas du tout 0 ──── 100 Très fortement |
| En ce moment, je me sens… anxieux.se | Pas du tout 0 ──── 100 Très fortement |
| **Most important event since the last beep** | |
| Pense maintenant à l’événement le plus important qui s’est passé depuis le dernier beep. |  |
| Cet événement t’était-il agréable ? | Très négativement ─── Très positivement |
| Comment as-tu réagi ? | ☐ J’ai pensé que je devais accepter que cela soit arrivé. ☐ J’ai souvent pensé à ce que je ressentais par rapport à ce que j’ai vécu. ☐ J’ai pensé que je pouvais tirer quelque chose de positif de la situation. ☐ J’ai pensé que la cause devait être en moi. ☐ J’ai pensé à quelque chose d’agréable plutôt qu’à ce qui s’était passé. ☐ J’ai pensé continuellement à quel point la situation avait été horrible. ☐ J’ai pensé que les autres étaient responsables de ce qui était arrivé. ☐ J’ai pensé à comment changer la situation. ☐ J’ai pensé que ce n’était pas si grave comparé à d’autres choses. |
| **Intra-interpersonal dynamic processes** | |
| En ce moment, je me sens… inutile | Pas du tout 0 ──── 100 Très fortement |
| En ce moment, je me sens… comme un fardeau pour les autres | Pas du tout 0 ──── 100 Très fortement |
| En ce moment, j’ai l’impression de ne pas faire partie d’un groupe | Pas du tout 0 ──── 100 Très fortement |
| En ce moment, je me sens… seul.e | Pas du tout 0 ──── 100 Très fortement |
| En ce moment, je sais qu’il y a des personnes avec qui je peux parler si je me sens mal. | Pas du tout 0 ──── 100 Très fortement |
| En ce moment, je me sens soutenu.e par quelqu’un qui comprend mes difficultés. | Pas du tout 0 ──── 100 Très fortement |
| En ce moment, je me sens… découragé.e par la vie | Pas du tout 0 ──── 100 Très fortement |
| En ce moment, je me sens… impuissant.e à changer les choses | Pas du tout 0 ──── 100 Très fortement |
| En ce moment, je ne vois aucune échappatoire à ce que je vis actuellement. | Pas du tout 0 ──── 100 Très fortement |
| En ce moment, je me sens piégé.e à l’intérieur de moi et j’aimerais m’éloigner de ce que je suis. | Pas du tout 0 ──── 100 Très fortement |
| En ce moment, je me sens… désespéré.e | Pas du tout 0 ──── 100 Très fortement |
| En ce moment, j’agis sans réfléchir aux conséquences. | Pas du tout 0 ──── 100 Très fortement |
| **Suicidal thoughts and behaviors** | |
| En ce moment, j’ai envie de me faire du mal, mais pas dans le but de mourir. | Pas du tout 0 ──── 100 Très fortement |
| En ce moment, j’ai l’impression que la vie ne vaut pas la peine d’être vécue. | Pas du tout 0 ──── 100 Très fortement |
| En ce moment, je pense à me suicider. | Pas du tout 0 ──── 100 Très fortement |
| Depuis le dernier beep, j’ai fait face à mes idées suicidaires en… | ☐ Parlant à un ami ou un pair ☐ Parlant à un parent ou un membre de la famille ☐ Parlant à un thérapeute, conseiller ou médecin ☐ Faisant quelque chose de relaxant ☐ Me distrayant avec autre chose ☐ Me disant quelque chose de calmant ou positif ☐ Pensant aux raisons de vivre ☐ Je n’ai pas eu de pensées suicidaires |
| Depuis le dernier beep, j’ai fait quelque chose pour me faire du mal, mais SANS vouloir mourir. | ☐ Oui ☐ Non |
| Depuis le dernier beep, j’ai tenté de me suicider. | ☐ Oui ☐ Non |

# Simulation Approach to Estimate Power in Multilevel Linear Models

Power and parameter-recovery simulations followed Lafit et al. [1] and were implemented with the PowerAnalysisIL Shiny application (<https://github.com/ginettelafit/PowerAnalysisIL>).

## Simulation procedure

More specifically, the simulation approach is based on the following steps:

1. Specify the multilevel model and its population parameters from theory, prior evidence, or pilot data.
2. Select a participant sample size and simulate a data set from the population model.
3. Fit the model and test each hypothesized fixed effect with a Wald test.
4. Repeat simulation and model fitting across the Monte Carlo replications.
5. Estimate power as the proportion of replications in which the null hypothesis is rejected.

## Effect of a level-1 continuous predictor (random slope)

Level 1: $Y_{it}=\gamma_{0i}+\gamma_{1i}X_{it}+\epsilon_{it}$

$$\gamma_{0i}=\beta_{00}+\nu_{0i}$$

$$\gamma_{1i}=\beta_{10}+\nu_{1i}$$

$Y_{it}$is the dependent variable

$X_{it}$is the level-1 predictor which is normally distributed $N(\mu_{X},\sigma_{X}^{2})$

$\epsilon_{it}$with autocorrelation $\rho_{\epsilon}$and variance $\sigma_{\epsilon}^{2}$

$\gamma_{0i}$is Gaussian distributed $N(0,\sigma_{\nu_{0}}^{2})$

$\gamma_{1i}$is Gaussian distributed $N(0,\sigma_{\nu_{1}}^{2})$

$$Cor(\nu_{0i},\nu_{1i})=\rho_{\nu_{01}}$$

$N$is the total number of individuals

$T$is the total number of repeated measurements for each individual

## Simulation setup

The simulations evaluated 40, 50, 60, 70, and 85 participants, with 56 repeated measurements per participant. Population values were 2.50 for the fixed intercept and 0.70 for the fixed within-person slope.

The level-1 error standard deviation was 0.20 with AR(1) autocorrelation of 0.33. The random-intercept and random-slope standard deviations were 3.00 and 0.15, respectively, with a correlation of 0.33.

The time-varying predictor had mean 1.70 and standard deviation 1.50 and was person-mean centered. Each condition used 1,000 Monte Carlo replications, a two-sided alpha of .05, and maximum-likelihood estimation.

Power, parameter recovery, and simulated outcome trajectories are summarized below.

**Figure 1.** Simulated power for the fixed intercept and fixed slope across participant sample sizes (N=40, 50, 60, 70, and 85), with 56 observations per participant.

**Table 1. Fixed-effect simulation results by participant sample size**

|  | **True value** | **Mean** | **Std.error** | **Bias** | **(1-alpha)% coverage** | **Power** |
| --- | --- | --- | --- | --- | --- | --- |
| Fixed intercept N 40 | 2.5 | 2.5240 | 0.0150 | 0.0240 | 0.935 | 0.999 |
| Fixed intercept N 50 | 2.5 | 2.4975 | 0.0131 | -0.0025 | 0.942 | 1.000 |
| Fixed intercept N 60 | 2.5 | 2.5074 | 0.0121 | 0.0074 | 0.942 | 1.000 |
| Fixed intercept N 70 | 2.5 | 2.5075 | 0.0114 | 0.0075 | 0.937 | 1.000 |
| Fixed intercept N 85 | 2.5 | 2.5047 | 0.0106 | 0.0047 | 0.937 | 1.000 |
| Fixed slope N 40 | 0.7 | 0.6995 | 0.0008 | -0.0005 | 0.936 | 1.000 |
| Fixed slope N 50 | 0.7 | 0.7009 | 0.0007 | 0.0009 | 0.939 | 1.000 |
| Fixed slope N 60 | 0.7 | 0.7007 | 0.0006 | 0.0007 | 0.947 | 1.000 |
| Fixed slope N 70 | 0.7 | 0.6999 | 0.0006 | -0.0001 | 0.941 | 1.000 |
| Fixed slope N 85 | 0.7 | 0.6996 | 0.0005 | -0.0004 | 0.955 | 1.000 |

*Note. Mean is the average estimate across replications; Std. error is the reported standard error; bias is the mean difference between the estimate and the population value; coverage is the proportion of confidence intervals containing the population value; and power is the proportion of rejected null hypotheses.*

**Table 2. Random-effect simulation results by participant sample size**

|  | **True value** | **Mean** | **Std.error** | **Bias** |
| --- | --- | --- | --- | --- |
| Standard deviation of the level-1 error N 40 | 0.20 | 0.2002 | 0.0150 | 0.0240 |
| Standard deviation of the level-1 error N 50 | 0.20 | 0.2001 | 0.0131 | -0.0025 |
| Standard deviation of the level-1 error N 60 | 0.20 | 0.2001 | 0.0121 | 0.0074 |
| Standard deviation of the level-1 error N 70 | 0.20 | 0.2001 | 0.0114 | 0.0075 |
| Standard deviation of the level-1 error N 85 | 0.20 | 0.2001 | 0.0106 | 0.0047 |
| Autocorrelation of the level-1 error N 40 | 0.33 | 0.3299 | 0.0007 | -0.0001 |
| Autocorrelation of the level-1 error N 50 | 0.33 | 0.3296 | 0.0006 | -0.0004 |
| Autocorrelation of the level-1 error N 60 | 0.33 | 0.3295 | 0.0005 | -0.0005 |
| Autocorrelation of the level-1 error N 70 | 0.33 | 0.3295 | 0.0005 | -0.0005 |
| Autocorrelation of the level-1 error N 85 | 0.33 | 0.3298 | 0.0005 | -0.0002 |
| Standard deviation of the random intercept N 40 | 3.00 | 2.9609 | 0.0106 | -0.0391 |
| Standard deviation of the random intercept N 50 | 3.00 | 2.9378 | 0.0099 | -0.0622 |
| Standard deviation of the random intercept N 60 | 3.00 | 2.9580 | 0.0086 | -0.0420 |
| Standard deviation of the random intercept N 70 | 3.00 | 2.9602 | 0.0080 | -0.0398 |
| Standard deviation of the random intercept N 85 | 3.00 | 2.9786 | 0.0071 | -0.0214 |
| Standard deviation of the random slope N 40 | 0.15 | 0.1473 | 0.0005 | -0.0027 |
| Standard deviation of the random slope N 50 | 0.15 | 0.1471 | 0.0005 | -0.0029 |
| Standard deviation of the random slope N 60 | 0.15 | 0.1476 | 0.0004 | -0.0024 |
| Standard deviation of the random slope N 70 | 0.15 | 0.1487 | 0.0004 | -0.0013 |
| Standard deviation of the random slope N 85 | 0.15 | 0.1485 | 0.0004 | -0.0015 |
| Correlation between the random intercept and the random slope N 40 | 0.33 | 0.3295 | 0.0046 | -0.0005 |
| Correlation between the random intercept and the random slope N 50 | 0.33 | 0.3182 | 0.0041 | -0.0118 |
| Correlation between the random intercept and the random slope N 60 | 0.33 | 0.3307 | 0.0037 | 0.0007 |
| Correlation between the random intercept and the random slope N 70 | 0.33 | 0.3333 | 0.0035 | 0.0033 |
| Correlation between the random intercept and the random slope N 85 | 0.33 | 0.3326 | 0.0030 | 0.0026 |

*Note. Mean is the average estimate across replications; Std. error is the reported standard error; and bias is the mean difference between the estimate and the population value.*

## Conclusion

Under the simulated conditions, 60 participants provide a reasonable basis for estimating random slopes: the random-slope standard deviation was recovered with small bias (0.1476 vs 0.1500; bias -0.0024), while fixed-slope power was 1.00 and coverage was 0.947. This conclusion is conditional on 56 observations per participant, the assumed effect sizes and covariance structure, and successful model estimation.

## Monte Carlo distributions

**Figure 2.** Monte Carlo distributions of the fixed- and random-effect estimates. Dashed vertical lines indicate the population values used to generate the data.

## Simulated outcome trajectories

**Figure 3.** Simulated outcome trajectory for the participant at the 5th percentile in the largest-sample condition (N=85). The dashed line indicates the participant-specific mean.

**Figure 4.** Simulated outcome trajectory for the participant at the 50th percentile in the largest-sample condition (N=85). The dashed line indicates the participant-specific mean.

**Figure 5.** Simulated outcome trajectory for the participant at the 95th percentile in the largest-sample condition (N=85). The dashed line indicates the participant-specific mean.

References:

1.	Lafit G, Adolf JK, Dejonckheere E, Myin-Germeys I, Viechtbauer W, Ceulemans E. Selection of the Number of Participants in Intensive Longitudinal Studies: A User-Friendly Shiny App and Tutorial for Performing Power Analysis in Multilevel Regression Models That Account for Temporal Dependencies. Adv Methods Pract Psychol Sci 2021 Jan;4(1):251524592097873. doi: 10/gjjxgd
